# Regulation of the MEI-1/MEI-2 Microtubule-Severing Katanin Complex in Early *Caenorhabditis elegans* Development

**DOI:** 10.1534/g3.116.031666

**Published:** 2016-08-12

**Authors:** Sarah M. Beard, Ryan B. Smit, Benjamin G. Chan, Paul. E. Mains

**Affiliations:** Department of Biochemistry and Molecular Biology, Alberta Children’s Hospital Research Institute, University of Calgary, T2N 4N1, Canada

**Keywords:** meiosis, spindle, microtubule severing, embryo, katanin

## Abstract

After fertilization, rapid changes of the *Caenorhabditis elegans* cytoskeleton occur in the transition from meiosis to mitosis, requiring precise regulation. The MEI-1/MEI-2 katanin microtubule-severing complex is essential for meiotic spindle formation but must be quickly inactivated to allow for proper formation of the mitotic spindle. MEI-1/MEI-2 inactivation is dependent on multiple redundant pathways. The primary pathway employs the MEL-26 substrate adaptor for the CUL-3/cullin-based E3 ubiquitin ligase, which targets MEI-1 for proteosomal degradation. Here, we used quantitative antibody staining to measure MEI-1 levels to determine how other genes implicated in MEI-1 regulation act relative to CUL-3/MEL-26. The anaphase-promoting complex/cyclosome, APC/C, the DYRK (Dual-specificity tyrosine-regulated kinase), MBK-2, and the CUL-2-based E3 ubiquitin ligase act together to degrade MEI-1, in parallel to MEL-26/CUL-3. CUL-2 is known to keep MEL-26 low during meiosis, so CUL-2 apparently changes its target from MEL-26 in meiosis to MEI-1 in mitosis. RFL-1, an activator of cullin E3 ubiquitin ligases, activates CUL-2 but not CUL-3 for MEI-1 elimination. HECD-1 (HECT/Homologous to the E6AP carboxyl terminus domain) E3 ligase acts as a MEI-1 activator in meiosis but functions as an inhibitor during mitosis, without affecting levels of MEI-1 or MEI-2. Our results highlight the multiple layers of MEI-1 regulation that are required during the switch from the meiotic to mitotic modes of cell division.

The cytoskeleton is a central player in maintaining cell structure, movement, shape, and cell division. Although the cytoskeleton itself has been extensively studied, less is known about what regulates cytoskeletal remodeling, especially during embryogenesis when cells rapidly adopt new forms and roles. Significant regulatory molecules can be in low abundance, short-lived, or transitory, making them difficult to identify and analyze. We use the nematode *Caenorhabditis elegans* as a model system to study the rapid transitions in the microtubule (MT) cytoskeleton in the early embryo. Fertilization triggers the completion of meiosis in the *C. elegans* zygote and within 15 min, the short, acentrosomal meiotic spindle must be disassembled and replaced by the astral mitotic spindle that fills most of the zygote’s volume (Albertson 1984; Kemphues *et al.* 1986; Albertson and Thomson 1993; McCarter *et al.* 1999; Yang *et al.* 2003). These dramatic changes require the precise regulation of molecules specific to each type of cell division. Protein degradation plays a key role in this meiosis-to-mitosis transition by eliminating oocyte-specific products that are no longer needed or would be detrimental to the developing embryo (Bowerman and Kurz 2006; Stitzel and Seydoux 2007; Verlhac *et al.* 2010; Robertson and Lin 2013).

The *C. elegans* genes *mei-1* and *mei-2* (meiosis defective) encode the two subunits of the katanin MT severing complex (Mains *et al.* 1990a; McNally and Vale 1993; Srayko *et al.* 2000; Roll-Mecak and McNally 2010; Sharp and Ross 2012). *mei-1* and *mei-2* are essential for meiotic spindle formation but must be rapidly inactivated prior to the first mitotic cleavage (Clark-Maguire and Mains 1994a). MEI-1/MEI-2 activity keeps meiotic spindles short, produces seeds for MT nucleation, and mediates the anaphase shortening of the spindle that is characteristic of the meiotic divisions (McNally *et al.* 2006, 2014; Srayko *et al.* 2006). Failure to eliminate katanin in mitosis results in lethality due to excess MT severing during the early cleavages (Mains *et al.* 1990a; Clark-Maguire and Mains 1994a; Srayko *et al.* 2006).

There are multiple redundant layers of regulation that ensure katanin MEI-1/MEI-2 MT severing is restricted to meiosis (Figure 1). The primary mode of MEI-1 regulation is through two independent protein degradation pathways. The major pathway involves MEL-26 (maternal effect lethal, Dow and Mains 1998) as a substrate recognition subunit that recruits MEI-1 to the CUL-3/cullin-based E3 ubiquitin ligase. This results in MEI-1 ubiquitylation and subsequent proteosomal degradation (Furukawa *et al.* 2003; Pintard *et al.* 2003b; Xu *et al.* 2003). Phosphorylation often precedes ubiquitylation and MBK-2 (mini-brain kinase), a member of the DYRK (Dual Specificity and Tyrosine Regulated Kinase) family of protein kinases, contributes to postmeiotic MEI-1 degradation (Pellettieri *et al.* 2003; Quintin *et al.* 2003; Ming Pang *et al.* 2004; Stitzel *et al.* 2006, 2007; Cheng *et al.* 2009; Wang *et al.* 2014). MBK-2 is not essential for MEL-26/CUL-3 degradation of MEI-1, but instead acts in a parallel protein degradation pathway with an unknown ubiquitin ligase (Lu and Mains 2007). RFL-1/ Uba3p (ectopic membrane ruffling) has also been implicated in MEI-1 degradation. *rfl-1* encodes a subunit of an E1 activating enzyme that leads to the addition of a ubiquitin-like Nedd8 to cullins that activates E3 ubiquitin ligase activity (Kurz *et al.* 2002; Pintard *et al.* 2003a; Sarikas *et al.* 2011). Other modes of MEI-1 regulation include the PP4 phosphatase complex that increases MT severing activity at anaphase II (Han *et al.* 2009; Gomes *et al.* 2013) and postmeiotic inhibition of *mei-1* mRNA translation by IFET-1/SPN-2 and OMA-1 (Li *et al.* 2009).

**Figure 1:**
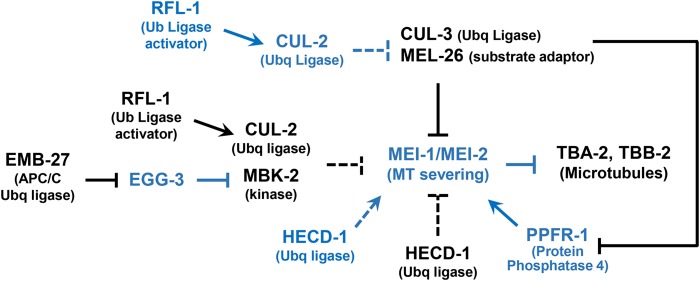
The MEI-1 regulatory pathway. Proteins active in meiosis are in blue and those active in mitosis are in black. During meiosis, the katanin MEI-1/MEI-2 complex is activated by PPFR-1 and HECD-1 and so severs microtubules (MTs). When meiosis is completed, CUL-3/MEL-26 increases and EMB-27/APC/C activates CUL-2/MBK-2, leading to MEI-1 degradation. CUL-2 also functions during meiosis to prevent premature MEL-26 accumulation. RFL-1 and HECD-1 function at both divisions. Dashed lines indicate that interactions may be indirect. Not shown are mitotic translational inhibition of MEI-1 mRNA (messenger RNA) by IFET-1/SPN-2 and OMA-1 or the other PP4 subunits that act with PPFR-1.

MEI-1 degradation is under temporal control to ensure that sufficient MT severing activity remains until the completion of meiosis. Levels of the MEI-1 inhibitor MEL-26 remain low until the completion of meiosis due to the activity of RFL-1 and another E3 ubiquitin ligase, which contains CUL-2 (Johnson *et al.* 2009). The MBK-2-mediated MEI-1 degradation pathway is activated upon the completion of meiosis by the anaphase promoting complex/cyclosome (APC/C) that both drives meiotic anaphase progression and degrades the MBK-2 inhibitor EGG-3 (Lu and Mains 2007; Stitzel *et al.* 2007; Cheng *et al.* 2009; Parry *et al.* 2009). APC/C might also target the katanin MEI-2 subunit as the latter has an APC/C consensus “destruction box” motif RxxLxxxxN (RkkLthakN in MEI-2) (Harper *et al.* 2002; Lu and Mains 2007). MEI-1 and MEI-2 are each dependent on the other for their expression (Clark-Maguire and Mains 1994a; Srayko *et al.* 2000).

Previous work showing that APC/C and MBK-2 act in parallel to MEL-26 was based on the logic that if two genes act in the same pathway, their mutations will not enhance (exacerbate) one another’s phenotypes in doubly mutant animals; once a mutation in one gene inactivates a pathway, loss of a second gene in the same linear pathway is irrelevant. In contrast, if two genes act in parallel, the double mutant shows a more extreme (*i.e.*, enhanced) phenotype than either of the single mutants. This analysis requires that at least one of the mutations must be a null; hypomorphic mutations acting sequentially may also show enhancement. Our previous work with MEL-26 and MBK-2 could rely on measuring embryonic viability because MEI-1 is the only essential substrate of MEL-26 (Lu *et al.* 2004; Lu and Mains 2005; Johnson *et al.* 2009) and so some *mel-26(null)* embryos survive at low temperatures due to the action of the MBK-2 pathway. In double mutants of *mel-26(null)* with a temperature-sensitive (*ts*) *mbk-2* allele at a semipermissive temperature all embryos die, demonstrating that the genes work in parallel. However, we could not order MBK-2, APC/C, and RFL-1 relative to each other because these genes have other essential targets and so their null phenotypes are lethal, precluding assays of hatching rates (Davis *et al.* 2002; Stitzel *et al.* 2006; Dorfman *et al.* 2009; Wang *et al.* 2014). In this work, we quantitatively measured anti-MEI-1 staining levels in double mutant strains as an alternative metric to embryonic viability. We show that APC/C and RFL-1 act in the MBK-2 pathway. We identified CUL-2 as the E3 ubiquitin ligase acting with MBK-2. In addition, we found that HECD-1, a HECT domain ubiquitin ligase (homologous to the E6AP carboxyl terminus), also regulates MEI-1 without apparent effects on MEI-1 protein levels. Unexpectedly, HECD-1 acts as a MEI-1 activator in meiosis but as an inhibitor in mitosis. Thus, the regulation of MEI-1 MT severing includes unanticipated layers of regulation.

## Materials and Methods

### Nematode culture and strains

All strains, including the wild type (N2, *var*. Bristol) were maintained under standard conditions at 15° unless otherwise stated (Brenner 1974). Hatching rates of complete broods of at least four hermaphrodites and > 400 offspring for each strain are reported as previously described (Mains *et al.* 1990b). *ts* strains were upshifted to their nonpermissive temperatures (20° or 25°) at the last larval stage (L4). The following alleles were used and detailed descriptions are found at http://www.wormbase.org. Linkage group *I*: *mei-1(ct46ts*, *ct46ct101*, and *or1178ts)*, *mei-2(ct98* and *sb39ts)*, and *mel-26(ct61sb4* and *or184ts)*. Linkage group *II*: *zyg-11(b2ts)* and *emb-27(g48ts)*. Linkage group *III*: *tbb-2(sb26)*, *cul-2(or209ts* and *ek1)*, *zer-1(gk165593)*, *rfl-1(or198ts)*, and *fem-2(b245ts)*. Linkage group *IV*: *hecd-1(ok1437)*, *mbk-2(dd5ts* and *pk1427)*, and *fem-1(hc17ts* and *e1965). mei-1(or1178ts)* included the transgenes *ruIs57[pAZ147 pie-1*::*GFP*::*tbb-2 unc-119(+)] III*; *ltIs37[Paa64:pie-1p*::*mCherry*::*his-58 unc-119(+)]. zer-1(gk165593)* is a nonsense allele isolated in the Million Mutant Project (Thompson *et al.* 2013). *zer-1* and *hecd-1* were outcrossed eight times before use and genotypes for strains that included these alleles were confirmed by PCR or sequencing. Alleles were often marked with closely-linked morphological markers to facilitate strain construction. The *tbb-2(sb26)*/β tubulin allele, which is refractory to MEI-1 severing, was included in all strains that were measured by anti-MEI-1 staining to restore normal embryo morphology for easier comparisons of cell stages (Lu *et al.* 2004).

### RNAi

RNAi feeding followed the protocol described by Kamath and Ahringer (2003). A pair of L4 hermaphrodites were placed together on RNAi plates and transferred every 24 hr or until they ceased to lay embryos. The first brood was not counted due to RNAi not being fully effective. The exception was for the *mbk-2(RNAi)* experiment, where RNAi effects take place earlier and so the first brood was included in the analysis. Control worms were fed with *Escherichia coli* carrying the L4440 vector plasmid.

### Antibody staining

Young adult or L4 hermaphrodites were upshifted to the appropriate temperature 24 hr before fixation. Mutant and wild-type gravid hermaphrodites were mounted on the same polylysine slide, far enough apart to accurately distinguish the two populations. Embryos were extruded from hermaphrodites with gentle pressure on the cover slip. The embryos were fixed with methanol-acetone and immediately used for antibody staining as described (Kemphues *et al.* 1986; Clark-Maguire and Mains 1994a). The same antibody solution covered both the wild-type and mutant samples on the same slide. Rabbit anti-MEI-1 (Clark-Maguire and Mains 1994a) and rabbit anti-PPFR-1 (Gomes *et al.* 2013) were used at a 1/100 dilution, rat anti-MEL-26 (Johnson *et al.* 2009) and rabbit anti-MEI-2 (Srayko *et al.* 2000) were used at a 1/50 dilution, and mouse anti-α-tubulin (M1A, Sigma-Aldrich Inc.) was used at a 1/200 dilution. FITC-conjugated goat anti-rabbit (Sigma-Aldrich Inc.) and Texas Red-conjugated donkey anti-mouse (Jackson ImmunoResearch Inc.) were used at a 1/100 dilution. Slides were mounted in an antifade solution (Roche Diagnostics) that contained 1 µg/ml DAPI (4’, 6-diamidine-2’phenylindole dihydrochloride, Roche) to visualize DNA.

### Measurement and analysis of anti-MEI-1 staining

Photomicrographs through a focal plane that included both centrosomes were taken on a Zeiss Axioplan microscope at 40 × magnification equipped with a Hamatsu Orca ER digital camera using constant settings and exposure time controlled with Zeiss Axiovision (4.7.1) software. Embryos were measured using ImageJ (http://imagej.nih.gov/ij/) by circling the perimeter of the embryo (or individual cells) and determining the mean pixel intensity. The polar body was excluded because it retains high levels of MEI-1 (Clark-Maguire and Mains 1994a). The traced area was superimposed to a nearby area in the photomicrograph free of embryos or worm debris and the pixel value was subtracted from the embryo value. All images presented here were cropped without further editing. Data for each experimental day was normalized to the average of the wild type for that day. Cells were scored as prophase up to the point where DAPI-stained chromosomes aligned at the middle of the tubulin-stained spindle, metaphase during the period of alignment, anaphase from the time when chromosomes separated until they reached the centrosome, and telophase/interphase until completion of cytokinesis. Statistical significance was assessed with the Mann–Whitney Rank Sum test using Sigma Plot software as some data sets did not show the normal distribution required for *t*-tests (paired *t*-tests led to the same conclusions in all cases).

### Cell cycle timing

Embryos were mounted on a 2% agarose pads (Sulston *et al.* 1983). Images were taken using the Zeiss Axioplan microscope equipped with Nomarski optics and a Hamatsu OrcaER digital camera with Axiovision software. The cell cycle times from the completion of one cytokinesis to the next for the first two mitotic cell divisions were determined from images taken every 20 sec in the central focal plane. P0 cycle length was measured from pronuclear fusion to when the cleavage furrow fully bisected the daughter cells.

### Data availability

Strains and reagents are available upon request. Additional immunostaining data are included in supplemental files.

## Results

### Quantification of anti-MEI-1 levels

Our previous work, which placed both APC/C and *mbk-2* in parallel to *mel-26*, took advantage of the viability of the null truncation allele *mel-26(ct61sb4)* (hereafter referred to as *mel-26(null)*) at 15° but not 25°. This allele behaves as a null in genetic tests at all temperatures but is inherently *ts*, indicating that the parallel MEI-1 degradation pathway is sufficient at 15° but not 25° (Dow and Mains 1998; Lu and Mains 2007). Thus, we could use hatching rates in double mutants with *mel-26(null)* to order genes in the pathway. However, we could not determine how APC/C and *mbk-2* acted relative to one another because their null alleles showed complete lethality due to their activity in other pathways. Therefore, we developed a quantitative method to measure anti-MEI-1 staining as a substitute for measuring enhancement based on viability. When MEI-1 is ectopically expressed in mitosis it localizes to the cytoplasm, with higher expression on the spindle and yet higher levels in the center of the centrosomes and on the chromosomes (Clark-Maguire and Mains 1994a). Previous work scored increases in ectopic MEI-1 as the ratio of centrosomal MEI-1 to that in adjacent cytoplasm free of MTs (Lu and Mains 2007). However, as centrosomal MEI-1 increases in mutants, MEI-1 also increases in the cytoplasm but they may not do so in parallel. Other reports have used a translation MEI-1::GFP fusion protein to measure MEI-1 expression (Pellettieri *et al.* 2003; Pintard *et al.* 2003b; McNally *et al.* 2006; Parry *et al.* 2009). However, the transgene used in those studies rescues *mei-1(null)* poorly (P. E. Mains, unpublished results) and crossing the transgene into many mutant backgrounds, each with multiple mutations, would be difficult. Therefore, we used antibody staining to measure MEI-1 levels.

**Figure 2:**
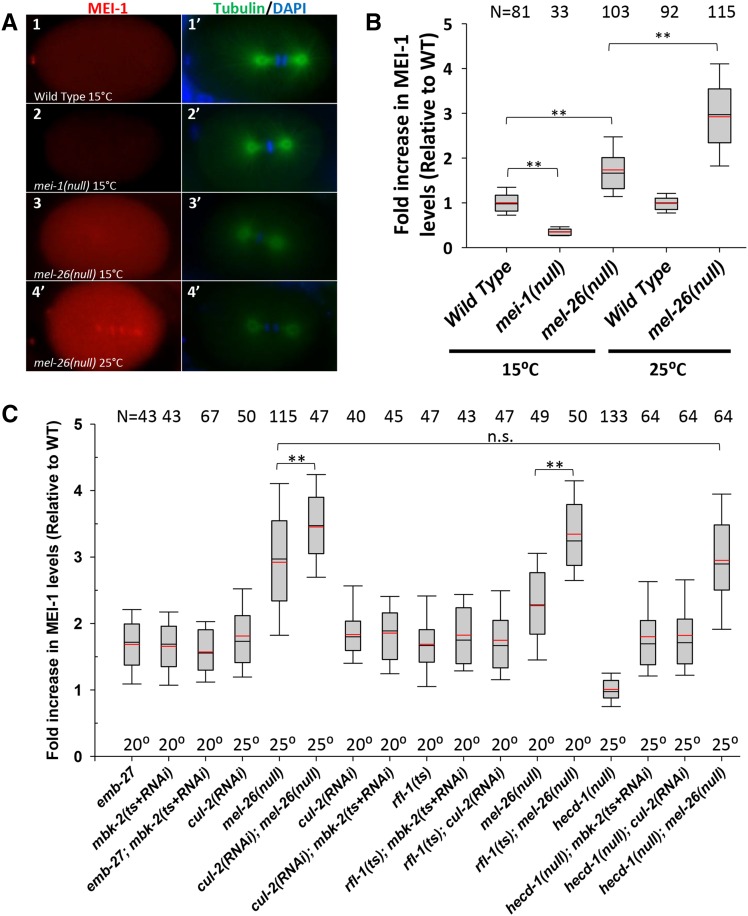
Quantitative anti-MEI-1 indirect immunofluorescence in wild-type and mutant embryos. All strains (including “Wild Type”) contained *tbb-2(sb26)* to restore normal morphology to embryos that expressed ectopic MEI-1 for easier comparisons. (A) Embryos at the first mitotic cleavage. Anti-MEI-1 (red) is shown in the left column with the corresponding anti-tubulin (green) and DAPI (blue) shown in the right column. The low levels of MEI-1 present in the wild type (A1 and A1’) were above levels shown in *mei-1(null)* embryos (A2 and A2’) at 15°, demonstrating antibody specificity. Higher than wild-type levels of MEI-1 were present in *mel-26(null)* at 15° (A3 and A3′) and levels further increased at 25° (A4 and A4’). (B) Anti-MEI-1 mean pixel intensity measurements were subtracted from the nearby background and divided by the average of the wild type processed on the same slide (1.0 = average wild-type level). Data are pooled for one-to-four-cell cell embryos and for all stages of the cell cycle (see Figure S1 for unpooled data). Boxes indicate the 25th to the 75th percentiles and whiskers show the 10th and 90th percentiles. Black lines indicate the median and red lines the mean. **: *P* < 0.001. N values are shown at the top of the chart. (C) Quantification of anti-MEI-1 levels for mutant strains. *emb-27*; *mbk-2* had similar levels of ectopic MEI-1 to the individual mutations, indicating the genes act in the same pathway. *cul-2*; *mel-26* had higher MEI-1 than the singles, indicating the genes act in parallel. *cul-2*; *mbk-2* was similar to the individual mutations, indicating these genes act in the same pathway. *rfl-1* double mutants with *mbk-2* or *cul-2* had MEI-1 levels similar to single mutants, and so these genes act in the same pathway. *rfl-1*; *mel-26* had elevated levels of MEI-1, and so those genes act in parallel. *hecd-1* had wild-type MEI-1 levels and *hecd-1* did not affect the levels found in *mbk-2*, *cul-2*, or *mel-26*, and so *hecd-1* does not appear to be involved in the degradation pathways. **, *P* ≤ 0.001; n.s., not significant; *P* ≥ 0.13. All other double mutant combinations did not differ significantly (0.13 > *P* > 0.90) from the higher of the corresponding single mutants, but this is not shown for to simplify this figure. N values are shown at the top of the chart and temperature are along the bottom. DAPI, 4’, 6-diamidine-2’phenylindole dihydrochloride; RNAi, RNA interference; WT, wild-type.

To optimize and standardize antibody staining methods, we measured the mean pixel intensity of MEI-1 staining within the entire embryo subtracted from the background. To minimize experimental variation, we normalized to the wild type processed on the same slide (see *Materials and Methods*). At 15°, *mel-26(null)* embryos showed approximately 1.5-fold higher anti-MEI-1 staining compared to wild-type embryos (*P* < 0.001) and this increased to 3 fold at 25° (*P* < 0.001) (Figure 2, A and B, Mann–Whitney Rank Sum tests are used throughout). Thus, our assay recapitulates the previous (more qualitative) results, showing that *mel-26(null)* results in ectopic MEI-1 and that this increases with temperature. The experiment was repeated on five different days, which demonstrated reproducibility (Supplemental Material, Figure S1A). Anti-MEI-1 staining was consistent for one cell (from the point when the female pronucleus became visible after meiosis II), two cell, and four cell embryos and did not differ with cell cycle (Figure S1, B and C), so the data we present pools all one-to-four-cell embryos. *mei-1(null)* embryos had significantly lower MEI-1 than wild-type (*P* < 0.001), confirming specificity of the antibody signal (Figure 2B). All measurements of ectopic MEI-1 staining were done in strains that included the *tbb-2(sb26)* allele (Lu *et al.* 2004; Lu and Mains 2007). This mutation encodes a β-tubulin that is refractory to MT severing and so restores normal embryo morphology to mutant strains with ectopic MEI-1, which otherwise have short, misoriented spindles and abnormal cytokinesis (Mains *et al.* 1990a). For simplicity, *tbb-2(sb26)* is left out of genotypes in most cases in the text and figures. This allele is not included in strains where embryo viability is scored, unless otherwise noted.

### The APC/C and MBK-2 act in the same pathway to regulate MEI-1 levels

Stitzel *et al.* (2007) demonstrated that the APC/C acts as an upstream activator of MBK-2 leading to MEI-1 degradation (Figure 1). It is possible that the APC/C also directly targets the destruction box consensus motif found in MEI-2, which in turn could lead to MEI-1 degradation. Indeed, *mei-2* mutations lead to the loss of MEI-1 staining (Clark-Maguire and Mains 1994a). This is also seen with the corresponding subunits of *Drosophila* katanin (Grode and Rogers 2015).

We asked if the APC-6/CDC16 subunit gene *emb-27* acts in parallel or strictly sequentially with *mbk-2*. This analysis requires double mutants, one of which must be null. If two genes act in parallel, they enhance one another’s phenotypes. The *ts* allele *emb-27(g48ts)* arrests embryos in meiosis I anaphase at the restrictive temperature (Golden *et al.* 2000), precluding later analysis of mitotically-expressed MEI-1. The null allele *mbk-2(pk1427)* in combination with *tbb-2(sb26)* shows defects in germline development and so hermaphrodites produce few oocytes (C. Lu and P. E. Mains, unpublished results), again preventing examination of MEI-1 levels during mitosis in early embryos. Therefore, we mimicked a null situation by combining the *ts* allele *mbk-2(dd5ts)* and *mbk-2(RNAi)*, both of which partly compromise *mbk-2* activity at the semipermissive temperature of 20°. *mbk-2(dd5ts)*; *tbb-2(sb26)* hermaphrodites were upshifted from 15° to 20° and placed on *mbk-2(RNAi)* bacteria as young adults, bypassing earlier *mbk-2*; *tbb-2(sb26)* defects in germline development. *mbk-2(dd5ts+RNAi)* had a hatching rate of 1/411 (the hatched embryo arrested without further growth), compared to 58% hatching for *mbk-2(dd5ts)* and 12% for *mbk-2(RNAi)* alone. Thus *mbk-2(dd5ts+RNAi)* represents a strong loss of function (*lf*) and was used to approximate a null. The *emb-27(g48ts)* allele is also partially compromised at 20°, showing 56% hatching (Lu and Mains 2007).

Ectopic MEI-1 was 1.7-fold higher in both *mbk-2(dd5ts+RNAi)* and *emb-27(ts)* embryos at 20° Compared to the wild type (Figure 2C). *emb-27*; *mbk-2(dd5ts+RNAi)* double mutants had ectopic MEI-1 staining levels of 1.6-fold above the wild type, not significantly different from the levels seen in each of the single mutants (*P* > 0.13). This lack of enhancement suggests that *emb-27*/APC/C only acts within the *mbk-2* pathway to induce MEI-1 degradation and does not have an additional function in parallel by targeting MEI-2.

### CUL-2 acts in parallel to MEL-26 to regulate MEI-1 levels

As we and others have reported previously (Stitzel *et al.* 2006; Johnson *et al.* 2009), loss of *cul-2* leads to ectopic MEI-1 expression in mitosis. Indeed, Kurz *et al.* (2002) observed that 2/9 *cul-2(RNAi)* embryos had *mel-26*-like phenotypes (short, misoriented spindles with abnormal cytokinesis). If CUL-2 is the E3 ubiquitin ligase acting in concert with MBK-2, in parallel to MEL-26 (Figure 1), then *mel-26*; *cul-2* would have higher MEI-1 levels than either of the single mutants. In contrast, no changes would be seen in *cul-2*; *mbk-2*. If CUL-2 is instead acting in the MEL-26/CUL-3 rather than the MBK-2 pathway, the *cul-2*; *mbk-2* genotype would show higher MEI-1 levels.

We initially used the *cul*-2*(ek1)* null allele and found ectopic MEI-1 staining in mitotic embryos; however, the null allele produced too few embryos for our analysis because of its earlier role in gonad development (Feng *et al.* 1999; Burger *et al.* 2013). As an alternative, we used *cul-2* RNAi on young adult worms or a *cul-2(ts)* allele to bypass the earlier defects in our *cul-2* experiments. MEI-1 levels in *cul-2(RNAi)* worms were 1.8-fold higher than the wild type at 25° (Figure 2C). *mel-26(null)* was 2.9-fold above the wild type. *mel-26(null)*; *cul-2(RNAi)* showed significantly higher (*P* < 0.001) ectopic MEI-1 staining than *mel-26(null)* alone, with levels reaching about 3.5-fold higher than the wild type. In contrast, at 20° there was no significant difference (*P* > 0.6) between the ectopic MEI-1 levels of *cul-2(RNAi)* (the higher of the two single mutants) and *cul-2(RNAi)*; *mbk-2(dd5ts+RNAi)* (Figure 2C). Thus, CUL-2 acts in parallel to CUL-3/MEL-26 and so could be the E3 ubiquitin ligase acting in concert with MBK-2.

### cul-2 genetic interactions indicate differing roles in meiosis and mitosis

We next asked if we could detect genetic interactions based on embryo viability that would reflect ectopic MEI-1 function in *cul-2* mutants. Interpreting *cul-2* double mutants with other *mei-1* pathway genes is complicated by the dual roles of *cul-2* in regulating both MEI-1 and delaying the build-up of the MEI-1 inhibitor MEL-26 until meiosis is complete (Johnson *et al.* 2009). However, analysis of *cul-2*; *mel-26(null)* is straightforward as this genotype eliminates effects on MEI-1 caused by premature increases in MEL-26. There was a strong genetic interaction between the *ts* allele of *cul-2(or209ts)* and the *mel-26(null)* allele at temperatures semipermissive for *cul-2*. At 15°, 64% of *cul-2(or209ts)* embryos hatched compared to 12% of *mel-26(null)* (Table 1, lines 1 and 2). If the two genes did not interact, then 64% × 12% = 7.7% of double mutant embryos should hatch. Instead, only 0.7% hatched, an 11-fold enhancement (line 3). This genetic interaction supports the conclusion from the anti-MEI-1 staining in Figure 2C that *cul-2* and *mel-26* work in parallel to eliminate MEI-1.

**Table 1:** Genetic interactions of MEI-1 pathway components

		Temperature								
		15°			20°			25°		
	Maternal Genotype*^a^*	% Hatch	Fold Change	% Male	% Hatch	Fold Change	% Male	% Hatch	Fold Change	% Male
1	*cul-2(ts)*	64		0.5	51		2.6			
2	*mel-26(null)*	12								
3	*cul-2(ts)*; *mel-26(null)*	0.7	11 × **↓**							
4	*tbb-2**^b^*	100		0						
5	*cul-2(ts) tbb-2*	40	1.6 × **↓**	2.6	27	1.9 × **↓**	6.0			
6	*rfl-1(ts)*	89			72					
7	*rfl-1(ts)*; *mel-26(null)*	0.9	12 × **↓**							
8	*mel-26(null)*; *tbb-2*	68	7.8 × ↑							
9	*rfl-1(ts)*; *tbb-2*	92								
10	*rfl-1(ts)*; *mel-26(null)*; *tbb-2*	65	77 × **↓**							
11	*mbk-2(ts)*	87			59					
12	*rfl-1(ts)*; *mbk-2(ts)*	35	2.2 × **↓**		0.5	85 × **↓**				
13	*mbk-2(ts)*; *tbb-2*				78					
14	*rfl-1(ts)*; *mbk-2(ts)*; *tbb-2*				1.5					
15	*hecd-1*	89			96		0	77		0
16	*hecd-1*; *mel-26(null)*	0^c^	> 190 × **↓**							
17	*hecd-1(RNAi)*	99			99					
18	*hecd-1(RNAi)*; *mel-26(null)*	0^c^	> 150 × **↓**							
19	*hecd-1*; *tbb-2*	91						85		0.4
20	*hecd-1(RNAi)*; *mel-26(null)*; *tbb-2*	50								
21	*mei-1(gf)*	12								
22	*hecd-1(RNAi)*; *mei-1(gf)*	0^c^	> 60 × **↓**							
23	*mbk-2(ts)*				74					
24	*hecd-1*; *mbk-2(ts)*				81					
25	*mei-2(sb39ts)*	78		1.3	41		2.2			
26	*hecd-1*; *mei-2(sb39ts)*	28	2.5 × **↓**	3.4	8	4.9 × **↓**	13			
27	*mei-2(ct98)*							71		2.6
28	*hecd-1*; *mei-2(ct98)*							19	2.9 × **↓**	8.9
29	*cul-2(ts)*; *hecd-1*	16	3.5 × **↓**	2.3	11	4.4 × **↓**	12			

a *ts* alleles are presumed hypomorphs at intermediate temperatures. *hecd-1 = ok1437, cul-2(ts) = or209, mbk-2(ts) = dd5, mei-1(ts) = or1178, mei-1(gf) = ct46, mel-26(null) = ct61sb4, mel-26(ts) = or184, rfl-1(ts) = or198*, and *tbb-2 = sb26*.

b Data from Lu *et al.* (2004).

c *N* values for lines 16, 18, and 22 are 1587, 1290, and 501, respectively. Fold enhancement is calculated based on if one embryo had hatched.

We examined the role of *cul-2* in meiosis and asked if premature MEL-26 expression in *cul-2(or209ts)* could in turn lower the amount of MEI-1 available for proper meiotic spindle formation and so cause meiotic defects. This situation would be equivalent to *mei-1* or *mei-2* hypomorphs, which result in meiotic nondisjunction due to spindle defects. While nondisjunction of autosomes results in lethality, if only the X chromosome is lost, viable XO males result (Him phenotype, high incidence of males). Thus, male progeny serve as an easily-scored metric of meiotic spindle defects (Mains *et al.* 1990a; Clandinin and Mains 1993; Lu and Mains 2005). The wild-type baseline frequency of XO males resulting from the selfing of XX hermaphrodites is 0.2% (Hodgkin *et al.* 1979). *cul-2(or209ts)* produced over 10-fold (2.6%) more males at 20°, indicating meiotic defects (Table 1, line 1). To determine if decreased MT severing was the cause of this Him phenotype, we compromised MEI-1 activity using the *tbb-2(sb26)* allele that encodes a β-tubulin refractory to MEI-1 severing. In addition to decreasing MT severing at mitosis (which restores normal morphology to embryos with ectopic mitotic MEI-1 as seen in Figure 2) (Lu *et al.* 2004; Lu and Mains 2005), this tubulin allele also decreases the normal MT severing that is required during meiosis: while *tbb-2(sb26)* alone does not increase the frequency of XO males, it does so in combination with *mei-2* hypomorphs (Lu *et al.* 2004). Indeed, the Him phenotype of *cul-2(or209ts)* was increased from the 2.6% described above to 6.0% with the addition of the tubulin allele at 20° (lines 1 and 5). At 15°, where *cul-2* alone had 0.5% males, *cul-2(or209ts) tbb-2(sb26)* hermaphrodites had 2.6% male offspring (line 5). These data with *tbb-2* indicate that MT severing is the relevant target for the *cul-2* Him phenotype. This is consistent with the loss of *cul-2* resulting in increased MEL-26, which in turn decreases MEI-1 activity during meiosis.

### rfl-1 acts upstream of CUL-2, but not MEL-26, to regulate MEI-1

RFL-1 is a component of an E1 complex that leads to ubiquitin ligase activation by coupling the Nedd8 ubiquitin-like molecule to cullins (Kurz *et al.* 2002; Pintard *et al.* 2003a; Petroski and Deshaies 2005; Sarikas *et al.* 2011). Like *mel-26* and *cul-2*, *rfl-1* has ectopic MEI-1 in mitosis (Kurz *et al.* 2002; Pintard *et al.* 2003a). To determine where RFL-1 is acting in MEI-1 regulation, we used the *ts* allele of *rfl-1(or198)* (Dorfman *et al.* 2009). *rfl-1* embryos showed 1.8-fold more MEI-1 staining than the wild type at the semipermissive temperature of 20° (Figure 2C). We found that there was no significant difference (*P* > 0.13) in ectopic MEI-1 staining levels between the single knockdowns, *rfl-1* and *mbk-2(ts+RNAi)*, and the double *rfl-1*; *mbk-2(ts+RNAi)*. In addition, *rfl-1*; *cul-2(RNAi)* showed no significant increase (*P* > 0.15) in ectopic MEI-1 staining compared to each single at 20°. This suggests that RFL-1 is acting in the same pathway as MBK-2 and CUL-2. In contrast, at 20°, *rfl-1*; *mel-26(null)* embryos did show significantly higher (*P* = 0.001) ectopic MEI-1 staining than *mel-26(null)* embryos (Figure 2C), indicating that *rfl-1* and *mel-26* likely act in parallel. Thus, the data indicate that RFL-1 appears to contribute to CUL-2-, but not CUL-3, -mediated MEI-1 degradation (Figure 1).

We examined hatching rates of double mutants between *rfl-1* and *mei-1* pathway mutations as another way to determine in which degradation pathway *rfl-1* functions. *rfl-1* strongly enhanced *mel-26(null)* lethality, leading to 12-fold less hatching than predicted if the genes acted independently (Table 1, lines 2, 6, and 7), supporting our conclusion that *rfl-1* acts in parallel to MEL-26. To determine if MT severing was the underlying cause of the enhanced lethality, rather than some other target shared by MEL-26 and RFL-1, we examined the effect of the *tbb-2(sb26)* allele that inhibits ectopic MEI-1 activity. As reported previously (Lu *et al.* 2004), *tbb-2(sb26)* suppresses *mel-26* lethality (lines 2 and 8). *tbb-2(sb26)* had no effect on *rfl-1* alone (line 9, Johnson *et al.* 2009), but *tbb-2(sb26)* did block the enhancement between *mel-26* and *rfl-1* (line 10). Thus, MEI-1/MT severing was the relevant target of the *rfl-1* genetic interaction with *mel-26*.

We next examined *rfl-1* genetic interactions with the *mbk-2* pathway. Unexpectedly, *rfl-1* also strongly enhanced *mbk-2* (85-fold at 20°; Table 1, lines 11 and 12). However, this lethality was not blocked by *tbb-2(sb26)* (lines 13 and 14) indicating that these genes likely share essential targets other than MEI-1. Indeed, while CUL-3 and MEL-26 may have only MEI-1 as their major target (Lu and Mains 2005, 2007), MBK-2, CUL-2, and RFL-1 have many other targets in the *C. elegans* germline and early embryo (Feng *et al.* 1999; DeRenzo *et al.* 2003; Pellettieri *et al.* 2003; Liu *et al.* 2004; Sonneville and Gonczy 2004; Sasagawa *et al.* 2005; Stitzel *et al.* 2006; Lu and Mains 2007; Dorfman *et al.* 2009; Guven-Ozkan *et al.* 2010; Starostina *et al.* 2010; Burger *et al.* 2013; Brockway *et al.* 2014; Wang *et al.* 2014).

### Candidate substrate recognition subunits used by CUL-2 leading to MEL-26 or MEI-1 inactivation

The CUL-2-containing E3 ubiquitin ligase apparently switches from targeting MEL-26 during meiosis to MEI-1 during mitosis (Figure 1). However, we do not know if either interaction reflects direct degradation or involves intermediates. Identification of the substrate recognition subunits used by CUL-2 for MEL-26 and MEI-1 regulation would help address these questions. Thus, we tested candidates for the substrate recognition subunits using the criteria that depleting a substrate adaptor targeting MEL-26 would suppress a *mel-26* hypomorph and enhance *mei-1* or *mei-2* hypomorphs. A substrate adaptor for MEI-1 (or MEI-2) would show the opposite interactions. In addition, these adaptors should alter the levels of MEI-1 or MEL-26. Follow-up testing for physical interactions between the substrate recognition subunits and MEI-1 pathway genes could then determine if CUL-2 targets each protein directly. However, none of the tested genes described below fulfilled our criteria.

In *C. elegans*, five proteins have been shown to bind CUL-2 as known or candidate substrate adaptors: FEM-1, ZYG-11, ZER-1, VHL-1, and LRR-1 (Starostina *et al.* 2007; Vasudevan *et al.* 2007; Merlet *et al.* 2010; Burger *et al.* 2013). We tested the first four for genetic interactions with *mel-26* and *mei-1* (*lrr-1* was not tested due to its sterility).

ZYG-11 acts with CUL-2 during meiotic progression (Liu *et al.* 2004; Sonneville and Gonczy 2004; Vasudevan *et al.* 2007). Double mutants between *zyg-11* and gain of function alleles of *mel-26* or *mei-1* have been shown previously to have minimal enhancement of embryonic lethality (Mains *et al.* 1990a). We also found no interactions between *zyg-11(ts)* and *lf* alleles of *mei-1*, *mei-2*, or *mel-26* (Table S1, lines 1–9). We previously reported that *zyg-11* does not affect MEL-26 accumulation (Johnson *et al.* 2009).

**Figure 3:**
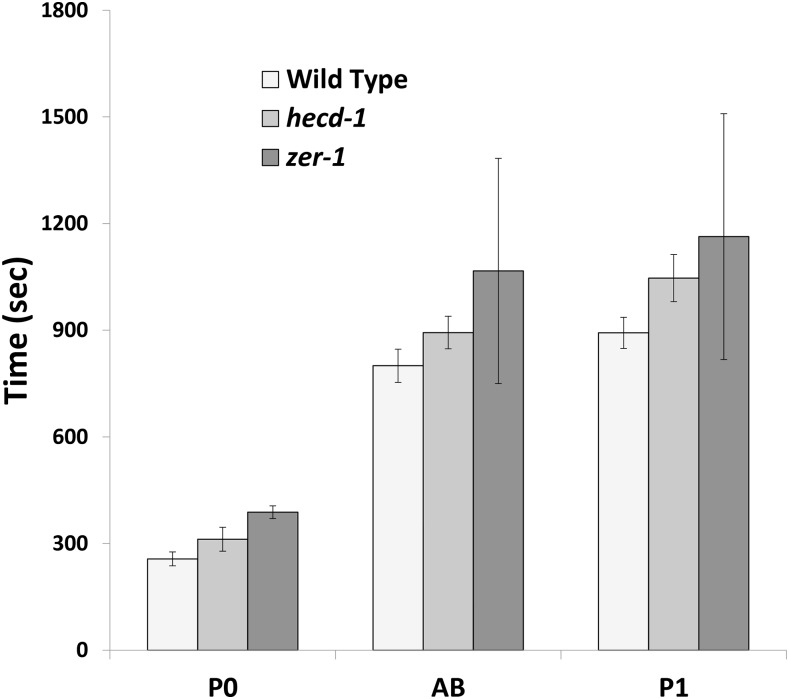
Cell cycles of *hecd-1* and *zer-1*. Embryos were observed by Nomarski microscopy at room temperature (∼23°) and the length of the cell cycles were noted. P0 cycle length was measured from pronuclear fusion to when the cleavage furrow fully bisected the daughter cells. The AB and P1 cell divisions were scored from one cleavage to the next. *hecd-1* and *zer-1* both lengthened cell cycles compared to wild-type for all three divisions (*P* < 0.04, unpaired *t*-tests, N ≥ 5 for each division of each genotype).

ZER-1 is a ZYG-11-related gene that binds CUL-2, but *zer-1* has no phenotype other than weakly enhancing *zyg-11* (Vasudevan *et al.* 2007). The *zer-1(gk165593)* allele (Q336stop in the 860 amino acid protein) suppressed *mel-26(null)* lethality. Additionally, *zer-1* also suppressed *mbk-2(ts)* (Table S1, lines 10–18). The suppression of both *mel-26(null)* and *mbk-2* by *zer-1* argue that *zer-1* is an activator of MEI-1, likely independent of those two pathways. However, *zer-1* showed little or no enhancement of *mei-1* or *mei-2* hypomorphs. MEI-1, MEI-2, and PPFR-1 staining of *zer-1* embryos did not show any obvious changes (Figure S2, A–L). Since previous work showed that *mbk-2(ts)* is suppressed by mutations that extend the cell cycle (Wang *et al.* 2014), we measured the lengths of the first two rounds of cleavage of *zer-1* embryos. Indeed, *zer-1* embryos have significantly longer cell cycle times (Figure 3). It is not clear if this alone explains the suppression of *mel-26*: as described below, *hecd-1* also has longer cell cycle times, but *hecd-1* enhanced rather than suppressed *mel-26* and *mei-1* defects.

FEM-1 acts with CUL-2 to target the TRA-1 sex determination transcription factor (Starostina *et al.* 2007) and the PAR-6 polarity protein in the early embryo (Pacquelet *et al.* 2008). The *ts* allele *fem-1(hc17ts)* showed the anticipated pattern of the substrate adapter for MEL-26 suppressing *mel-26(ts)* while enhancing *mei-1(ts)*, although both interactions were modest (Table S1, lines 19–27). However, the allele used, *hc17ts*, is an unusual *fem-1* mutation in affecting only the gonad and not the soma (Hodgkin 1986). The *fem-1* null allele, *e1965*, failed to recapitulate the genetic interactions of *hc17ts*. Furthermore, while FEM-1 acts with FEM-2 in regulating TRA-1 degradation, we found no genetic interactions with *ts* or null alleles of *fem-2* (Table S1, lines 28–33). Finally, *fem-1* did not affect MEL-26 accumulation as would be anticipated for a MEL-26 substrate adapter (Figure S2, M–P).

*vhl-1* is involved in *C. elegans* DNA repair and longevity (Muller *et al.* 2009; Sendoel *et al.* 2010). However, *vhl-1(RNAi)* had no genetic interactions with *mel-26(null)* or *mel-26(ts)* (Table S1, lines 34–36).

### hecd-1 enhances mel-26(null) but does not affect MEI-1 levels

In an RNAi screen for ubiquitin ligases acting in parallel to MEL-26, we identified HECD-1, a HECT domain (Homologous to the E6-AP Carboxyl Terminus) E3 ubiquitin ligase, the ortholog of human Hectd1 (Chen and Greenwald 2014). A null allele *hecd-1(ok1437)* (Chen and Greenwald 2014) and *hecd-1(RNAi)* showed little effect on viability on their own but both decreased embryonic viability of *mel-26(null)* mutants, from 12 to 0% (*N* = 1587 and 1290, respectively), and so enhancement was at least 190-fold (Table 1, lines 2 and 15–18). *tbb-2(sb26)* rescued *mel-26*; *hecd-1(RNAi)* lethality (lines 19 and 20), indicating that MTs are the relevant target for the *hecd-1* genetic interactions. This interpretation was confirmed by showing that a *mei-1(ct46gf)* allele, which is refractory to MEL-26-mediated degradation (Clark-Maguire and Mains 1994a; Pintard *et al.* 2003b), was also enhanced by *hecd-1(RNAi)* (lines 21 and 22). In contrast, *hecd-1* did not affect *mbk-2* lethality (lines 23 and 24). Together, these results indicate that HECD-1 is an inhibitor of MEI-1 function, acting in parallel to MEL-26.

Loss of a MEI-1 inhibitor could suppress hypomorphic MEI-1 activity. Unexpectedly, *hecd-1* instead showed a two-to-five-fold enhancement of the lethality of two different *mei-2* hypomorphs. This lethality was accompanied by an increase in the percentage of XO males among the surviving progeny (Table 1, lines 15 and 25–28): *hecd-1* alone did not increase the level of spontaneous males, but there was a sixfold increase in male progeny for *mei-2(sb39ts)*; *hecd-1* at 20° and 3.5-fold for *mei-2(ct98)*; *hecd-1* at 25°. The increase in male progeny (likely via meiotic nondisjunction) suggests that the enhancement is due to meiotic spindle defects rather than nonspecific interactions leading to lethality. Thus, HECD-1 is acting as an activator of MEI-1 function at meiosis but as an inhibitor in mitosis (Figure 1). Finally, *hecd-1* also enhanced *cul-2* lethality, and this was accompanied by a fivefold increase in male progeny (Table 1, lines 1, 15, and 29).

We hypothesized that HECD-1 contributes to the degradation of mitotic MEI-1 in parallel to MEL-26. However, quantitative staining of *hecd-1* embryos at 25° showed wild-type MEI-1 in mitosis (Figure 2C and Figure S3, A–D). Furthermore, *hecd-1* did not affect MEI-1 levels (*P* > 0.4) in sensitized backgrounds that included *mbk-2(dd5ts+RNAi)*, *cul-2*, or *mel-26* (Figure 2C). HECD-1 could also influence regulators of MEI-1 activity, but we found no obvious changes in MEI-2, MEL-26, or the MEI-1 activator PPFR-1 by immunostaining (Figure S3, E–P).

**Figure 4:**
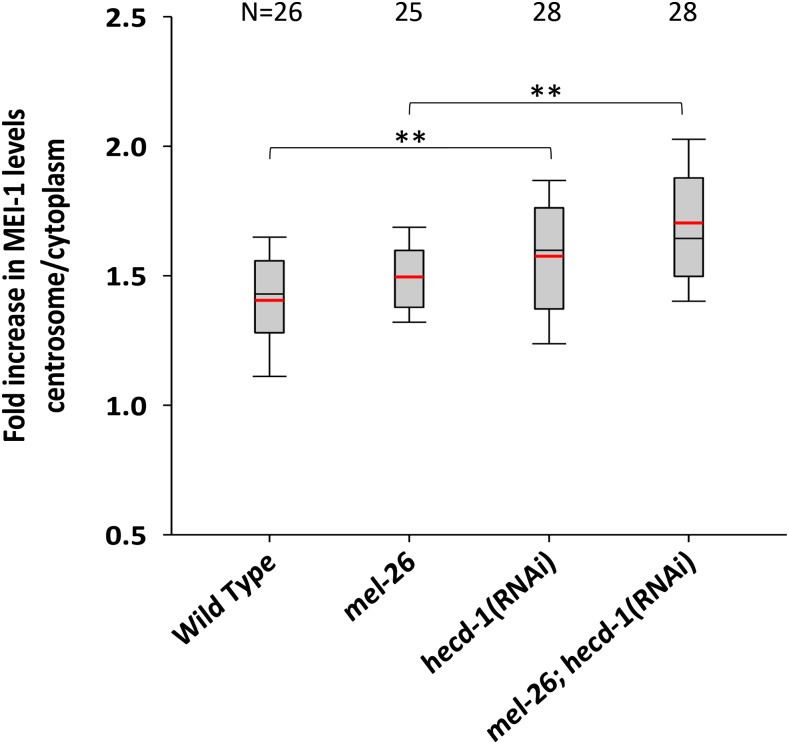
Quantification of centrosomal anti-MEI-1 levels. The MEI-1 level at the centrosome was compared to the embryo as a whole in wild-type, *mel-26*, and *hecd-1* embryos at 25°. Boxes indicate the 25th to the 75th percentiles and whiskers show the 10th and 90th percentiles. Black lines are at the median and red lines at the mean. *hecd-1(RNAi)* increased the ratio of centrosome/embryo relative to wild-type and also increased the ratio in the presence of *mel-26(null). mel-26* strains included the suppressor mutation *tbb-2(sb26)* to restore normal embryo morphology. **: *P* ≤ 0.003. N values are shown at the top of the figure. Note that unlike Figure 2, 1.0 does not represent the wild-type measurement. RNAi, RNA interference.

HECT domain proteins often alter the localization or trafficking of their targets rather than leading to proteosomal degradation (Mukhopadhyay and Riezman 2007; Metzger *et al.* 2012; Sarkar and Zohn 2012; Sato *et al.* 2014). The immunostaining results imply that HECD-1 is not influencing MEI-1 degradation. To test the effect of HECD-1 on MEI-1 location, we measured the ratio of anti-MEI-1 staining at the centrosome compared to the embryo as a whole. The ratio increased from an average of 1.5 in *mel-26* to 1.7 in *mel-26*; *hecd-1* (Figure 4). Although modest, this was statistically significant (*P* < 0.001). However, it is not clear that this small change alone accounts for the strong genetic interactions.

We examined the effects of *hecd-1* on the cell cycle. One possibility is that *hecd-1* shortens the cell cycle to decrease the ability of other compromised MEI-1 regulators to complete their function, which might explain the genetic enhancement with MEI-1 pathway genes. However, *hecd-1* had the opposite effect, lengthening the cell cycle (Figure 3). Both *hecd-1* and *zer-1* lengthen the cell cycle, but have opposite genetic interactions with *mel-26*.

## Discussion

At fertilization, the *C. elegans* embryo rapidly transitions from the meiotic to mitotic divisions (Albertson 1984; Kemphues *et al.* 1986; McCarter *et al.* 1999; Yang *et al.* 2003). Meiosis-specific products, such as the MEI-1/MEI-2 katanin MT severing complex, must be downregulated or eliminated as their persistence would be detrimental to later development (Clark-Maguire and Mains 1994a; Bowerman and Kurz 2006; Stitzel and Seydoux 2007; Verlhac *et al.* 2010; Robertson and Lin 2013). By measuring genetic interactions using quantitative antibody staining and embryo hatching rates, we determined how APC/C, MBK-2, CUL-2, RFL-1, and HECD-1 function relative to one another (Figure 1). Some genetic interactions as measured by embryo viability were modest, sometimes in the range of only twofold from that predicted assuming independence. These could be misleading and might represent nonspecific effects on general viability. Therefore, we focused on genetic interactions likely to be specific to the MEI-1 pathway: those that also increase meiotic nondisjunction, a characteristic of hypomorphic MEI-1 function, or interactions that were modified by a tubulin allele that specifically affects MEI-1-mediated MT severing.

### APC/C and MBK-2 act sequentially to degrade MEI-1 in mitosis

APC/C and MBK-2 were previously shown to act in parallel to CUL-3/MEL-26 for MEI-1 degradation (Lu and Mains 2007). While APC/C is required for the activation of MBK-2 to trigger MEI-1 degradation at meiotic exit (Maruyama *et al.* 2007; Stitzel *et al.* 2007), it was possible that APC/C could also act directly on the MEI-1/MEI-2 complex in a third pathway. Indeed, MEI-2 has an APC/C consensus destruction box (D-box). Here, we have demonstrated that APC/C and MBK-2 act strictly sequentially with one another and in parallel with MEL-26: double mutants of *emb-27* (APC-6/CDC16) and *mbk-2* showed the same level of anti-MEI-1 staining as the single mutants, demonstrating that the genes act in the same pathway. In contrast, mutations of the two genes increased MEI-1 levels when each were combined with *mel-26*, demonstrating that *emb-27* and *mbk-2* act in parallel with *mel-26* (Figure 2).

Gomes *et al.* (2013) showed that mutating the MBK-2 phosphorylation site on MEI-1 affects MEL-26 interactions with MEI-1 in yeast two-hybrid assays. This may imply that MBK-2 is also acting within the MEL-26/CUL-3 pathway in addition to our evidence that MBK-2 and MEL-26 act in parallel. However, this predicts that the *mbk-2* single mutant would have MEI-1 levels higher than the loss of *mel-26* alone, which was not observed (Figure 2). Possibilities to explain the lack of a genetic requirement of *mbk-2* for *mel-26* function is that another kinase phosphorylates the same MEI-1 residue as does MBK-2, or that *in vivo* interactions between unphosphorylated MEI-1 and MEL-26 are sufficiently strong to mediate MEI-1 degradation.

### CUL-2 regulates MT severing by multiple pathways

Given that MBK-2 is acting in parallel to the MEL-26/CUL-3 E3 ubiquitin ligase, we asked if the CUL-2 E3 ubiquitin ligase was acting in concert with MBK-2. Kurz *et al.* (2002) reported a weakly penetrant *cul-2(RNAi)* phenotype of small, misoriented spindles typical of *cul-3* and *mel-26*. Previous work found that MEI-1 persists into mitosis when CUL-2 was inactivated (Stitzel *et al.* 2006; Johnson *et al.* 2009). Our quantitative staining showed that CUL-2 acts with MBK-2, in parallel to MEL-26: *cul-2(RNAi)* increases MEI-1 levels in *mel-26* but not *mbk-2* backgrounds (Figure 2). The genetic enhancement of *mel-26* lethality by *cul-2* also suggests that the two genes act in parallel (Table 1).

CUL-2 acts at two points of MEI-1 regulation, having opposite effects at meiosis and mitosis (Figure 1). MEL-26 levels are low during meiosis but then increase. When *cul-2* is lost, MEL-26 accumulates prematurely to high levels during meiosis (although mitotic levels are not increased) (Johnson *et al.* 2009). We asked if high meiotic MEL-26 in *cul-2* mutants resulted in the meiotic defects due to lowered MEI-activity. Meiotic spindle defects stemming from suboptimal MEI-1 activity result in a high incidence of males (Him) phenotype due to nondisjunction generating XO males from XX hermaphrodite oocytes (Hodgkin *et al.* 1979; Clandinin and Mains 1993). Notably, at semipermissive temperatures, *cul-2(ts)* has the Him phenotype. It is possible that this nondisjunction phenotype could arise from the delayed meiotic progression seen in *cul-2* mutants (Liu *et al.* 2004; Sonneville and Gonczy 2004) rather than MEI-1 misregulation. However, the *cul-2* Him phenotype was likely mediated through compromised MEI-1 function as males were further increased when *cul-2* was combined with the tubulin mutation *tbb-2(sb26)* (Lu *et al.* 2004) that hinders MEI-1 activity (Table 1). The allele *mei-1(ct46ct103)* shows a similar mixture of both meiotic and mitotic defects to those of *cul-2*. This mutation decreases but does not eliminate MEI-1 enzymatic function, resulting in a meiotic Him phenotype, but the product persists into mitosis with sufficient activity to cause mitotic phenotypes (Clandinin and Mains 1993; Clark-Maguire and Mains 1994b; McNally and McNally 2011; McNally *et al.* 2014).

CUL-2 targets many substrates in the germline and acquires additional substrates in the newly fertilized embryo (Feng *et al.* 1999; DeRenzo *et al.* 2003; Liu *et al.* 2004; Sonneville and Gonczy 2004; Pacquelet *et al.* 2008; Merlet *et al.* 2010; Starostina *et al.* 2010). We do not know if CUL-2 regulation of MEL-26 or MEI-1 is direct and so we tried to identify the substrate adapters that could mediate the genetic interactions. Unlike mutations of *cul-2* that both increase MEI-1 activity in mitosis while decreasing MEI-1 in meiosis (via ectopic MEL-26), the loss of a substrate adapter would most likely affect meiosis or mitosis, but not both. Thus an adaptor would likely have reciprocal interactions with hypomorphs of *mel-26* *vs.* *mei-1* or *mei-2*. Unfortunately, none of the candidates tested met these genetic criteria (Table S1) or altered levels of MEI-1, MEI-2, MEL-26, and/or PPFR-1 (Figure S2).

### RFL-1 activates the MBK-2/CUL-2-mediated MEI-1 degradation pathway

RFL-1 leads to the neddylation of cullins, activating these E3 ubiquitin ligases (Kurz *et al.* 2002; Pintard *et al.* 2003a; Bosu *et al.* 2010; Sarikas *et al.* 2011; Pierce *et al.* 2013). RFL-1 is involved in neddylation of both *C. elegans* CUL-2 and CUL-3 (Luke-Glaser *et al.* 2007; Dorfman *et al.* 2009; Bosu *et al.* 2010). RFL-1 appears to function with CUL-2 to temporally regulate MEL-26 meiotic levels (Johnson *et al.* 2009). During mitosis, we found that RFL-1 acts sequentially with the MBK-2/CUL-2 pathway and in parallel to MEL-26-mediated MEI-1 degradation; loss of *rfl-1* further increased MEI-1 levels when combined with *mel-26* but did not do so in combination with *mbk-2* or *cul-2(RNAi)* (Figure 2). *rfl-1* enhancement of *mel-26* lethality also indicates that the genes act in parallel and this was ameliorated by *tbb-2(sb26)*, demonstrating that MEI-1 is a relevant target (Table 1). *rfl-1* also enhanced lethality in the parallel *mbk-2* pathway, which would imply that RFL-1 acts in both MEI-1 degradation pathways. However, *rfl-1*; *mbk-2* enhanced lethality did not stem from MEI-1 misregulation since MEI-1 levels were not increased in the double mutant and lethality was not suppressed by *tbb-2(sb26)* (Figure 2 and Table 1). Furthermore, if RFL-1 acted in both pathways, *rfl-1* mutants alone would have very high levels of ectopic MEI-1, which was not the case (Figure 2). Thus, *mbk-2* and *rfl-1* must act in parallel on other targets to explain the enhancement of lethality. Many targets other than MEI-1 have been reported for MBK-2 and RFL-1 (Feng *et al.* 1999; DeRenzo *et al.* 2003; Pellettieri *et al.* 2003; Liu *et al.* 2004; Sonneville and Gonczy 2004; Sasagawa *et al.* 2005; Stitzel *et al.* 2006; Lu and Mains 2007; Dorfman *et al.* 2009; Guven-Ozkan *et al.* 2010; Starostina *et al.* 2010; Burger *et al.* 2013; Brockway *et al.* 2014; Wang *et al.* 2014).

Previous reports assumed that the effects of RFL-1 on MEI-1 levels were mediated through CUL-3/MEL-26 rather than CUL-2 (Kurz *et al.* 2002, 2005; Pintard *et al.* 2003a; Dorfman *et al.* 2009). *rfl-1* mutants affect neddylation of both CUL-2 and CUL-3 (Luke-Glaser *et al.* 2007; Dorfman *et al.* 2009; Bosu *et al.* 2010). Neddylation/deneddylation cycles are proposed to activate Cullin complexes by mediating the exchange of substrate adaptors (Pierce *et al.* 2013). Since most *cul-3(RNAi)* lethality is suppressed by *tbb-2(sb26)* (Johnson *et al.* 2009), MEI-1 may be the major essential CUL-3 target and so dynamic exchange of CUL-3 substrate adapters might not be critical to the *C. elegans* embryo. Meanwhile, CUL-2 may require RFL-1 for MEI-1 degradation to facilitate exchange of the substrate adaptors for other CUL-2 targets in the germline and embryo (Feng *et al.* 1999; DeRenzo *et al.* 2003; Liu *et al.* 2004; Sonneville and Gonczy 2004; Sasagawa *et al.* 2005; Dorfman *et al.* 2009; Guven-Ozkan *et al.* 2010; Starostina *et al.* 2010; Burger *et al.* 2013; Brockway *et al.* 2014). In addition to MEI-1 degradation, RFL-1 appears to also function with CUL-2 by maintaining low meiotic MEL-26 levels for timely progression through meiosis (Johnson *et al.* 2009).

### HECD-1 acts in MEI-1 regulation without affecting protein levels

We found that HECD-1 genetically acts as both a MEI-1 activator in meiosis (*hecd-1* enhanced *mei-2* hypomorphs) and a MEI-1 inhibitor in mitosis (*hecd-1* enhanced mutations that result in ectopic MEI-1, Table 1). Both interactions appeared specific to MEI-1 regulation, as *hecd-1* increased *mei-2* meiotic nondisjunction (as indicated by XO male offspring) and *hecd-1* enhancement of *mel-26* was suppressed by *tbb-2(sb26). hecd-1* increased *mel-26* lethality by > 190-fold, the strongest enhancement we have observed in the *mei-1* system. However, *hecd-1* had no discernible effects on protein levels of MEI-1, MEI-2, or PPFR-1 (Figure 2 and Figure S3).

HECTD E3 ubiquitin ligases differ from cullin-based E3 ligases (*e.g.*, worm CUL-2/3*)* in that they directly bind the substrate without an adapter subunit and can build ubiquitin chains via K63 instead of K48 linkages (Mukhopadhyay and Riezman 2007; Metzger *et al.* 2012). K63 ubiquitin modifies protein location, protein–protein interactions, and/or trafficking, although cases of target degradation are known (Li *et al.* 2013; Segref *et al.* 2014). HECTD E3 ligases function in mammalian neural tube formation, regulate heat shock protein 90 secretion and subsequent cell migration (Sarkar and Zohn 2012), placental development (Sarkar *et al.* 2014), and Wnt signaling (Tran *et al.* 2013). *C. elegans hecd-1* acts in Notch signaling (Chen and Greenwald 2014), vulva development (Cui *et al.* 2006), mitochondrial metabolism (Segref *et al.* 2014), and in membrane protein endocytosis after fertilization (Sato *et al.* 2014).

Although we did not observe any change in MEI-1, MEI-2, or PPFR-1 levels in *hecd-1* mutants, effects could be subtle. A speculative model is that HECD-1 modifies MEI-1 or MEI-2 to increase function in the meiotic relative to the mitotic spindles. Thus, *hecd-1* loss would shift the balance back toward mitotic MEI-1 function. This would explain why *hecd-1* enhances mutants resulting in both meiotic loss and mitotic gain of MEI-1. We did find that *hecd-1* increased MEI-1 centrosomal localization relative to the cytoplasm (Figure 4), but the effects were modest and it is not clear if they are sufficient to explain our genetic interactions. Further studies will focus on identifying HECD-1 targets.

In conclusion, our results indicate that many levels of regulation are involved in ensuring that MEI-1 is quickly inactivated before mitosis to allow for proper embryogenesis. We showed that the APC/C acts strictly sequentially with MBK-2. We demonstrated a role for CUL-2 acting in concert with MBK-2 to degrade MEI-1. The neddylation pathway gene *rfl-1* regulates MEI-1 through CUL-2 rather than CUL-3. Finally, we identified a role for HECD-1 in regulating MEI-1 at both meiosis and mitosis, apparently independent of protein levels. Further analysis of these players are needed to decipher their exact roles in the process.

## 

## Supplementary Material

Supplemental Material
