# Supplementary material for: Regulation of the MEI-1/MEI-2 Microtubule-Severing Katanin Complex in Early Caenorhabditis elegans Development
Source: G3 (Bethesda). 2016 Aug 12;6(10):3257–68. doi: 10.1534/g3.116.031666 (PMC5068946; doi:10.1534/g3.116.031666)
Supplement: Supplemental Material [file supp_6_10_3257__index.html]

Regulation of the MEI-1/MEI-2 Microtubule-Severing Katanin Complex in Early Caenorhabditis elegans Development — Supplemental Material 

# Regulation of the MEI-1/MEI-2 Microtubule-Severing Katanin Complex in Early *Caenorhabditis elegans* Development

## Supplemental Material for Beard, *et al*, 2016

**Files in this Data Supplement:**

- Table S1 - Genetic interactions of MEI-1 pathway components with possible CUL-2 substrate recognition subunits. (.pdf, 45 KB)
- Figure S1 - Consistency of anti-MEI1 staining of data that are summed for Figure 2B. (.pdf, 154 KB)
- Figure S2 - The genes encoding CUL-2 substrate adapters *zer-1* and *fem-1* do not alter expression of MEI-1 pathway genes. (.pdf, 372 KB)
- Figure S3 - *hecd-1* does not alter expression of MEI-1 pathway genes. (.pdf, 349 KB)
