# Supplementary material for: Regulation of the MEI-1/MEI-2 Microtubule-Severing Katanin Complex in Early Caenorhabditis elegans Development
Source: G3 (Bethesda). 2016 Aug 12;6(10):3257–68. doi: 10.1534/g3.116.031666 (PMC5068946; doi:10.1534/g3.116.031666)
Supplement: Supplemental Material [file supp_g3.116.031666_TableS1.pdf]

Table S1. Genetic interactions of MEI-1 pathway components with possible CUL-2 substrate recognition subunits

	Maternal Genotype [a]	15º		20º		25º	
		% Hatch	Fold Change	% Hatch	Fold Change	% Hatch	Fold Change
1	*mel-26(null)*	14		0.3		0	
2	*zyg-11(ts)*	90		70			
3	*zyg-11(ts); mel-26(null)*	8.1	1.6x ↓				
4	*mel-26(ts)*	49		8.0			
5	*zyg-11(ts); mel-26(ts)*	32	1.5x ↓				
6	*mei-1(ts)* [b]	97		94		1.3	
7	*zyg-11(ts); mei-1(ts)*			55	1.2x ↓		
8	*mei-2(ts)*			48			
9	*zyg-11(ts); mei-2(ts)*			50			
10	*zer-1*	97		97		80	
11	*zer-1; mel-26(null)*	26	1.9x ↑	6.6	22x ↑	0.2	
12	*zer-1; mel-26(ts)*	45		13	1.7x ↑	0	
13	*zer-1; mei-1(ts)*					4.4	3.4x ↑
14	*mei-1(gf)*	23		3.6			
15	*zer-1; mei-1(gf)*	28		4.0			
16	*zer-1; mei-2(sb39)*			58			
17	*mbk-2(ts)*			51		3.7	
18	*zer-1; mbk-2(ts)*			69	1.4x ↑	29	8x ↑
19	*fem-1(ts)*			98		97	
20	*fem-1(ts); mel-26(ts)*			22	2.8x ↑		
21	*fem-1(ts); mel-26(null)*	11		3.1	10x ↑		
22	*fem-1(ts); mei-1(ts)*	77	1.2x ↓	54	1.7x ↓		
23	*fem-1(null, m+z-)*			98			
24	*fem-1(null, m+z-); mel-26(ts)*	45					
25	*fem-1(null, m-z-); mel-26(ts)*	30 [c]					
26	*fem-1(null, m-z+); mei-1(ts)* [d]			81			
27	*fem-1(null, m-z+); mei-1(ts)* [d]			85			

28	*fem-2(ts)*			98	
29	*fem-2(ts); mel-26(ts)*	25	2x ↓		
30	*fem-2(ts); mei-1(ts)*			94	2.3
31	*fem-2(null, m+z-)*			98	
32	*fem-2(null, m+z-); mel-26(null)*	4.0	2x ↓		
33	*fem-2(null, m+z-); mei-1(ts)*			90	
34	*vhl-1(RNAi)*			100	
35	*vhl-1(RNAi); mel-26(ts)*	64 [e]		40 [e]	
36	*vhl-1(RNAi); mel-26(null)*	14 [f]		2.6 [f]	

[a] *ts* alleles are presumed hypomorphs at intermediate temperatures. *fem-1(ts) = hc17, fem-1(null) = e1965, fem-2(ts) = b245, fem-2(null) = e2105, mbk-2(ts) = dd5, mei-1(ts) = or1178, mei-1(gf) = ct46, mei-2(ts) = sb39, mel-26(null) = ct61sb4, mel-26(ts) = or184, zer-1 = gk165593, zyg-11(ts) = b2.*

[b] From Table 1.

[c] N=111, all other values >400.

[d] These are the *m+z-* progeny of *m-z-* females crossed to wild-type males. All other genotypes in the Table are maternal genotypes.

[e] vector control = 63% and 29% hatching at 15° and 20° respectively. The host bacteria (HT115) grows into much thicker lawns than OP50 and better nutrition may result in higher control hatching levels. Alternatively, it is more difficult to score unhatched embryos in the thicker HT115 lawns, which could lead to an underestimation of hatching rates.

[f] vector control = 31% and 0.8% hatching at 15° and 20° respectively.
