# Supplementary material for: Regulation of the MEI-1/MEI-2 Microtubule-Severing Katanin Complex in Early Caenorhabditis elegans Development
Source: G3 (Bethesda). 2016 Aug 12;6(10):3257–68. doi: 10.1534/g3.116.031666 (PMC5068946; doi:10.1534/g3.116.031666)
Supplement: Supplemental Material [file supp_g3.116.031666_FigureS1.pdf.html]

 G3: Genes | Genomes | Genetics 

New Article Format Available: Genome Report

## Current Issue : December 2016

## Early Online: December 2, 2016

- ### Select an Issue from the Archive

  June 2011 - December 2016
- ### Search for Articles

  June 2011 - December 2016

For an alternate route to G3: Genes | Genomes | Genetics use this URL:  http://intl.g3journal.org  [More Information]
