# Supplementary material for: Regulation of the MEI-1/MEI-2 Microtubule-Severing Katanin Complex in Early Caenorhabditis elegans Development
Source: G3 (Bethesda). 2016 Aug 12;6(10):3257–68. doi: 10.1534/g3.116.031666 (PMC5068946; doi:10.1534/g3.116.031666)
Supplement: Supplemental Material [file supp_g3.116.031666_FigureS2.pdf]

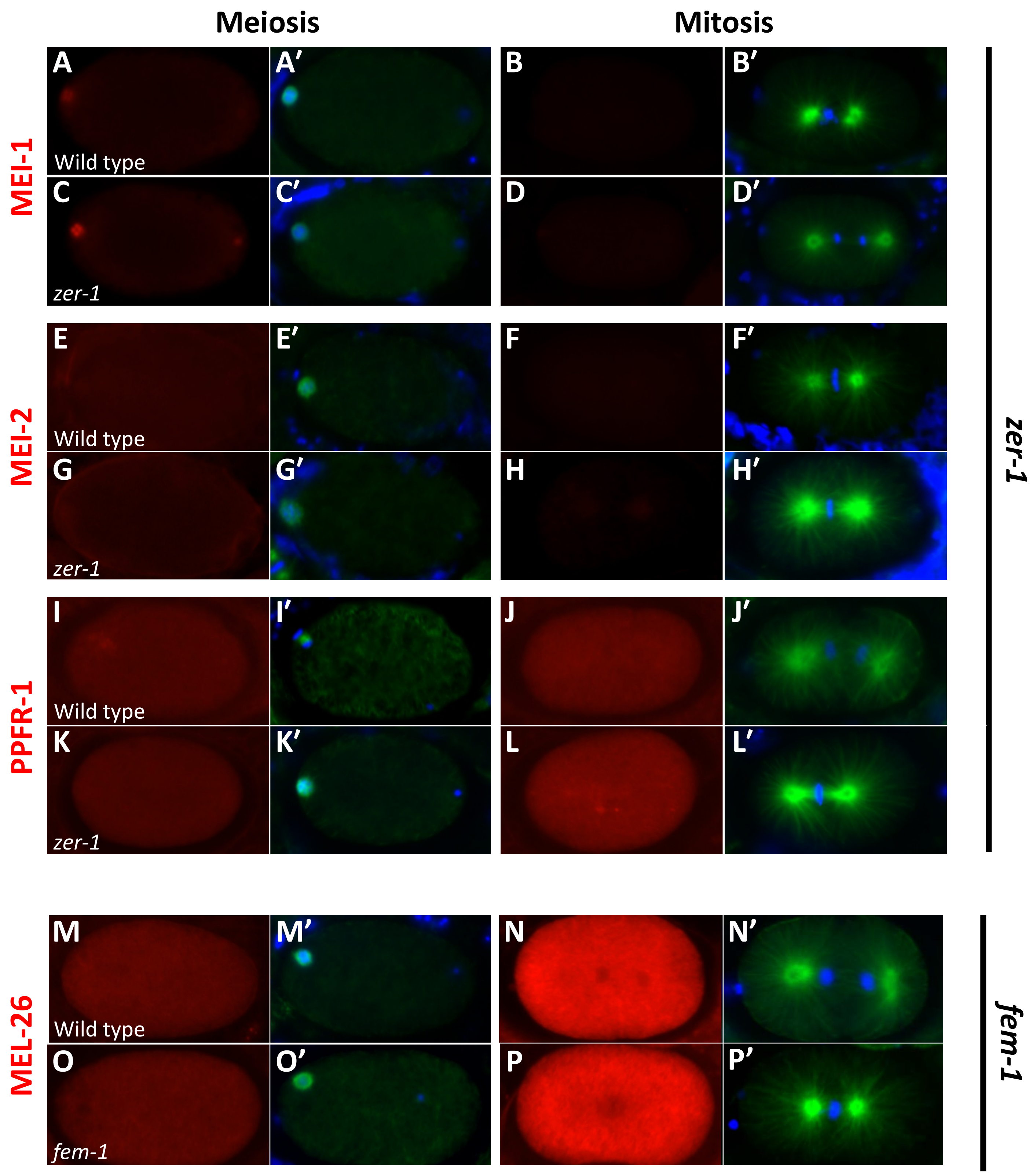

Meiosis
Mitosis
MEI-1
MEI-2
PPFR-1
MEL-26
A
A′
B
B′
Wild type
C
C′
D
D′
zer-1
E
E′
F
F′
Wild type
G
G′
H
H′
zer-1
I
I′
J
J′
Wild type
K
K′
L
L′
zer-1
M
M′
N
N′
Wild type
O
O′
P
P′
fem-1
zer-1
fem-1

Figure S2

Figure S2. The genes encoding CUL-2 substrate adapters *zer-1* and *fem-1* do not alter expression of MEI-1 pathway genes. Wild-type and *zer-1* embryos were stained with antibodies directed against MEI-1 (A-D), MEI-2 (E-H) and PPFR-1 (I-L), which are shown in red. The corresponding images marked with prime letters show DAPI (blue) and anti-tubulin (green) in the same embryos. The left pairs are in meiosis and the right pairs are in mitosis. *fem-1(hc17)* did not alter levels of MEL-26 (M-P). Because MEL-26 levels increase with meiotic progression, embryos were synchronized at meiosis I using *emb-27(ts)* at the restrictive temperature for 2 hours prior to staining as described previously (JOHNSON *et al.* 2009), thus "wild type" is *emb-27* and "*fem-1*" is *fem-1; emb-27.*
