# Supplementary material for: Regulation of the MEI-1/MEI-2 Microtubule-Severing Katanin Complex in Early Caenorhabditis elegans Development
Source: G3 (Bethesda). 2016 Aug 12;6(10):3257–68. doi: 10.1534/g3.116.031666 (PMC5068946; doi:10.1534/g3.116.031666)
Supplement: Supplemental Material [file supp_g3.116.031666_FigureS3.pdf]

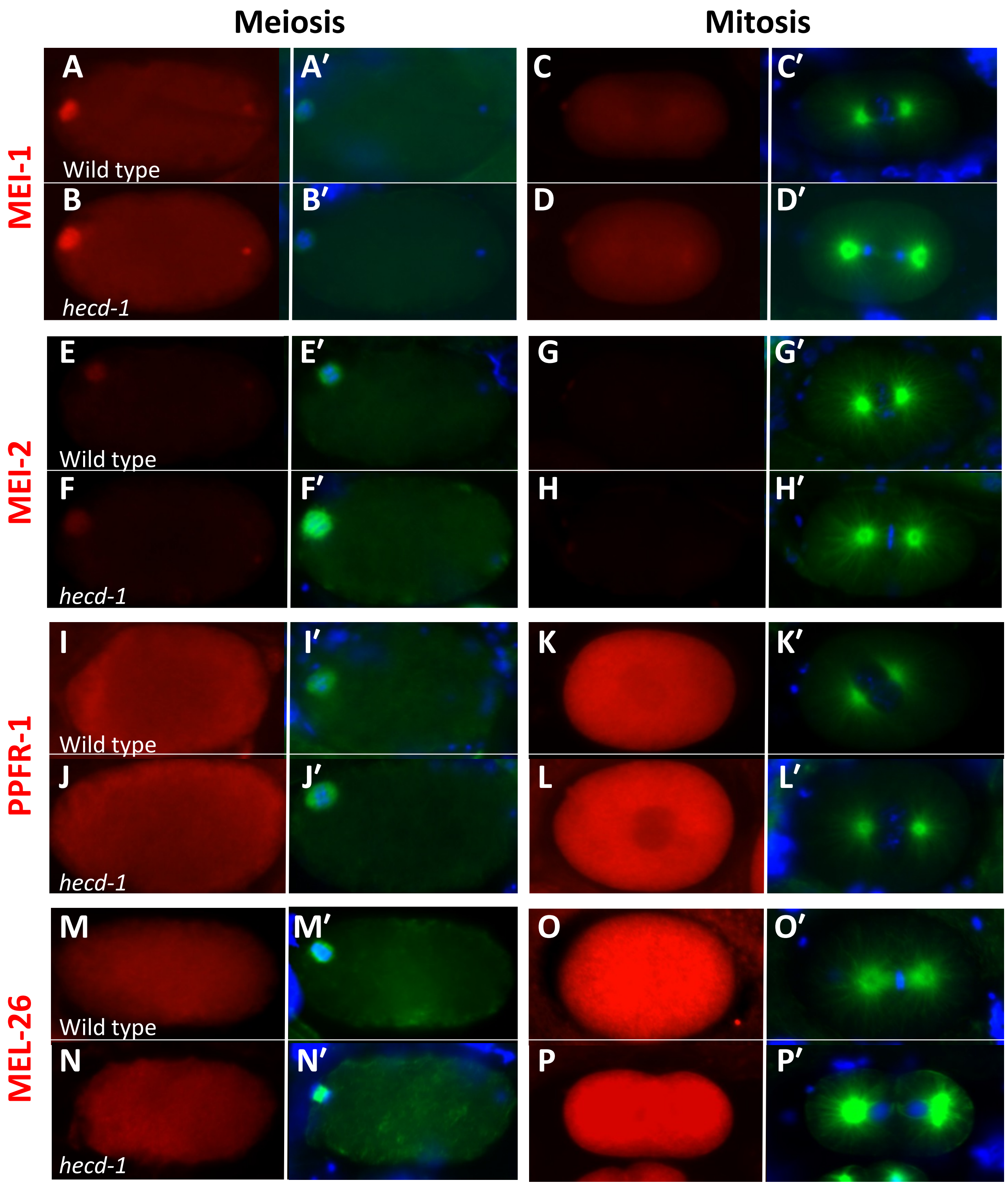
Meiosis
Mitosis
MEI-1
MEI-2
PPFR-1
MEL-26
A
A′
C
C′
Wild type
B
B′
D
D′
hecd-1
E
E′
G
G′
Wild type
F
F′
H
H′
hecd-1
I
I′
K
K′
Wild type
J
J′
L
L′
hecd-1
M
M′
O
O′
Wild type
N
N′
P
P′
hecd-1

Figure S3

Figure S3. *hecd-1* does not alter expression of MEI-1 pathway genes. Wild-type and *hecd-1* embryos were stained with antibodies directed against MEI-1 (A-D), MEI-2 (E-H), PPFR-1 (I-L) and MEL-26 (M-P), which are shown in red. The corresponding images marked with prime letters show DAPI (blue) and anti-tubulin (green) in the same embryos. The left pairs are in meiosis and the right pairs are in mitosis. No obvious differences in any genotypes were observed for *hecd-1* beyond changes in relative centrosomal MEI-1 noted in Figure 3.
